# Acknowledgment to Reviewers of *Gels* in 2021

**DOI:** 10.3390/gels8020077

**Published:** 2022-01-26

**Authors:** Gels Editorial Office

**Affiliations:** MDPI AG, St. Alban-Anlage 66, 4052 Basel, Switzerland

Rigorous peer-reviews are the basis of high-quality academic publishing. Thanks to the great efforts of our reviewers, *Gels* was able to maintain its standards for the high quality of its published papers. Thanks to the contribution of our reviewers, in 2021, the median time to first decision was 15 days and the median time to publication was 34 days. The editors would like to extend their gratitude and recognition to the following reviewers for their precious time and dedication, regardless of whether the papers they reviewed were finally published:
Aaron AlfordKun HuangAbdelhafed TalebKun LiangAbdulrahman A. BalhaddadKun XueÁdám JuhászKunhao YuAdarsh Nayarassery NarayananKunyan SuiAdéla MelcrováKwan Hyi LeeAdina SantanaKyohei FukataAfang ZhangKyoko KofujiAgnes KlarLacramioara PopaAgnieszka Gadomska-GajadhurLan JiangAgnieszka KowalczykLarisa TsarkovaAgnieszka Kulawik-PióroLaura ChronopoulouAgnieszka PilarskaLauro BucioAgnieszka Sobczak-KupiecLei NieÁgota TóthLeila RoufegarinejadAhmad KoohiLi NaAhmed OrabiLibor MatejkaAkiko ToyotamaLidija GradišnikAkitaka ItoLih-Sheng TurngAlastair N. CormackLin QiuAlberto De IseppiLingxiao XieAlberto FraileLiqing ZhaoAlberto García-PeñasLuca BurrattiAlcilene Rodrigues MonteiroLucia BaldinoAldo NicosiaLucia CatucciAleksey VishnyakovLucimara G. De La TorreAlena SalákováLuis Felipe De Paula SantosAlena VimmrováLuis García-FernándezAlessandro PezzellaLuis RodrigoAlessio BucciarelliLuqman Ali ShahAlexander MarrasLuying XunAlexander ShcherbakovMaciej JarzebskiAlexandros ChremosMaciej SkotakAlexey L. IordanskiiMaciej SzwastAli AyatiMagdalini D. TitirlaAli KhanjariMahboubeh YeganehAli M. AlqahtaniMahdi BodaghiAli OladMahsa SalehiAli Sedaghat DoostMahuya DasAli Shaygan NiaMakoto TakafujiAli TamayolMallesh KurakulaAltaf Hussain KanharManisha PandeyAlwar RamaniManuel DescoAlyne Lamy-MendesManuel PiñeroAmérica Chávez-MartinezManuela Maria IftimeAmjad BalangeMarc C. A. StuartAna AlcudiaMarc SchmutzAna Cláudia SantosMarcel KrzanAna Corte-RealMarcelino ManeiroAna Luisa Martínez-LópezMarek KieliszekAna Maria PutzMaria De La Luz Reyes-VegaAna-Maria PutzMaria Grazia RaucciAnastassia MashentsevaMaria ManconiAnatoly ZinchenkoMaria MarudovaAnca DinischiotuMaria MolinaAndré LopesMaria P. SokolovaAndrea AtreiMaría Sancho-TelloAndrea BuneaMaria Valentina DinuAndrea Luca TascaMariana E. GhicaAndreea PușcașMarija Gizdavic-NikolaidisAndreea-Teodora IacobMarija M. BabićAndres AyuelaMarin MicutzAndrew GravelleMarino LavorgnaAndrew PadalhinMario GrassiAndrey DrozdovMariorosario MasulloAndriati NingrumMarta GrochowiczAngelo PutignanoMarta TunesiAni BaghdasaryanMartin InAnirudh SharmaMartina ŠídlováAnja EngelMary Beth Browning MonroeAnka Trajkovska PetkoskaMarzena StefaniukAnna FlorowskaMasamichi YamanakaAnna Maria OsyczkaMassimo MarielloAnna ShipovskayaMatthew S. TurnerAnna ŻbikowskaMauro ValenteAnna Zimoch-KorzyckaMayra Aguilar-ZárateAnnalisa DalmoroMayya UspenskayaAnnarita StringaroMazen NazalAnthony HarkerMd. Abul AzadAntonella AccardoMeenakshi DuttAntonietta S. QuiggMehrez E. El-NagarAntonio Fernández-BarberoMeiwen CaoAnugya BhattMei-Yu YehAnuj KumarMelike FirlakAnurodh TripathiMesut UyanerApurba DasMiao DuArchana Bhaw-LuximonMichael DunneAris GiannakasMichael GradzielskiArkadi ArinsteinMichael T. CookArkadii ArinsteinMichaela Schulz-SiegmundArkadiusz JózefczakMichail KastelloriziosArthur D. TinocoMichał BielejewskiArtur TurekMichele MarinoAshveen NandMiklós NagyAurel DiaconMilan StankovicAvathamsa AthirasalaMinghao YaoAyesha MurtazaMinhao XieAznan IsmailMirna Habuda-StanicBabatunde OkesolaMiroslava Dušková-SmrčkováBahareh AzimiMiruna StanBapan PramanikMiryam Criado GonzalezBarkat KhanMitsuhiro ShibayamaBeata Anna ZasonskaMitsumasa TaguchiBehrouz GhoraniMizuho KondoBenoit LoppinetMohammad Afsar UddinBhawana ThakurMohammad Javed AnsariBimal JanaMohammad Mirazul IslamBing XuMohammadreza ShokouhimehrBingbing GaoMohammed EmranBjørn Torger StokkeMohammed F. HamzaBong Geun ChungMohammed HawashBorsa JuditMohammed Salah NasrBoštjan ViharMohd BuraidahBouchta SahraouiMônica Feijó NaccacheBożena KarolewiczMonica OrellanaBożena TyliszczakMonika GoseckaBruno Henrique VilsinskiMuhammad AdeelC. K. AbdullahMuhammad JamilC. PatrickiosMuhammad MujtabaCai YuhangMurugesan ChandrasekaranCan ZhangMuthuchamy NallalCarlo CastellanoMyunghwan ByunCarlo DiaferiaNader ShehataCarlo GonzatoNader Taheri-QazviniCarlos Garcia-GonzalesNadir BettacheCarmen PirasNaofumi NagaCaroline BorderonNaphtali A. O’ConnorCaroline R. SzczepanskiNarendar DudhipalaCarson J. BrunsNathalie MignetCatalin I. PruncuNayan Ranjan SinghaCatalin TutaNeil ClarkeCatalin ZahariaNela BibireCécile EchalierNhamo ChaukuraCésar C. Leyva-PorrasNicolas MuzzioCesare CamettiNikita KuznetsovChaenyung ChaNikolai VatinChangsik SongNikolaos A. VainosChen LiNileshkumar DubeyChen XieNimer MurshidChengying BaiNirzari GuptaCheol-Hee AhnNishant ChakravortyChi Bum AhnNonappa NonappaChihching ChungNouf Faisal AlharbyChii-Rong YangNunzio TuccittoChinmay SatamNurhasni HasanChi-Ping LiOctavian BuiuChirag GodiyaOleksandr TomchukChristian DemitriOlga A. MostovayaChristina TangOlga Warszawik-HendzelChristopher J. WilsonOmar AzzaroniChristopher Stephen Andrew MusgraveOmid AkhlaghiChristopher V. HughesOrnella UrsiniClassius Ferreira Da SilvaOswaldo BaffaCláudia PellizzonOzgul GokClaudia TomasiniP. Abdul AzeemConcepción ValenciaPablo Di ChennaCorneliu CojocaruPaolo BoffanoCoro EcheverriaPasqualina GalloCristian PetcuPatricia Lopez-SanchezCristina ChircovPatrícia NicolucciCristina OrbeciPatricio SantagapitaCuixia SunPatrick C.L. HuiDaisuke AsaiPatrycja Domalik-PyzikDamien DupinPaula M. FerreiraDana AkilbekovaPaula Malo De MolinaDaniel Fernández-QuirozPaula OssowiczDaniel Hermida-MerinoPaulo H. S. PiccianiDaniela FrankePavel GurikovDarinka ChristovaPaweł GrochDario PasiniPayam ZarrintajDarius MilčiusPedro FernandesDavid A. SchiraldiPedzisai MakoniDavid Onoja PatrickPer HanssonDavid SchiraldiPeter KasakDawang ZhangPeter SantschiDa-Wei HuangPetra Obioma NnamaniDebasish HaldarPetri MurtoDebora CamposPhung LeDeepankar Kumar AshishPierre WeissDemetra GiuriPim Walace Doti DoDemin CaiPiyanut PinyouDeng-Guang YuPlamen KirilovDi JiaPonnusamy PalanisamyDimitris KioupisPrachi TharejaDirk KucklingPradipta Ranjan RautaDishary BanerjeePratheep Kumar AnnamalaiDmitriy BerilloPriyadarshi ChakrabortyDolores Remedios SerranoProkopios GeorgopanosDominique Larrea-WachtendorffQian YeDonghee SonQiang LiuDorota Kołbuk-KoniecznyQilin YuDursun SaraydınQin XuDuyoung ChoiQixian ChenE. JosephQuansan YangEcaterina Stela DraganQufu WeiEduardo GuzmánRachel LetteriEhsan Nazarzadeh ZareRadu MitrutEhsan ZeimaranRafal KaminskiEkaterina PrikhozhdenkoRaffaele PuglieseEkaterina ZhulinaRajendran JC BoseElena DamiáRajesh KumarElena ShchelokovaRakkiyappan ChandranElena StoccoRaluca NicuEleonora SocoRami K. SuleimanElham AhmadianRanjit DeElham Hosseini NejadRaquel P. F. GuinéElisabete Pereira Dos SantosRati NayakElisabeth GillRattapol PinnaratipElodie GuilminotRaviraj M. KulkarniElsa GalbisRebeca HernandezEmilia P. CollarRen-Jei ChungEmilija FriganovićRichard ArmEmilio BucioRichard D’ArcyEmin YılmazRichard HoogenboomEneko LarranetaRichard WeissEric FreyssingeasRita De ZorziEsam Bashir YahyaRobert LuxenhoferEsam YahyaRobert W. Huigens IIIEtienne DucrotRoberta AngeliniEugenio GarribbaRoberta G. ToroEunice CarrilhoRocio Yaneli Aguirre LoredoEva PinhoRoksana MarkiewiczEwa JakubczykRolando BarbucciFabio DomeniciRomána ZelkóFacundo MatteaRongcong LuoFang QianRuei-Feng ShiuFang-Yi LinRuhuai MeiFarnaz BatoolRui TamuraFatemeh MokhtariRuimeng ZhangFatih PuzaRun-Sheng LinFatmanur Tuǧcu-DemirözRupert KarglFayaz AliRusso EleonoraFei LiuRüstem KeçiliFei XuSabino Aurelio BufoFelicity HanSai SagiriFelix PlamperSajjad Sayyar RoudsariFeng ChenSalim AlbukhatyFeng LiSalvatore GalloFernando Notario-PérezSalvatore MarulloFerry MelchelsSalvatore VerreFilip DyčkaSamantha C. PinhoFilippo RossiSamarendra MajiFiora ArtusioSameera ShafiFiore Pasquale NicolettaSantiago GrijalvoFioretta AsaroSantosh GhoshFlavio RizzolioSara PourshahrestaniFlorin IordacheSarah GlaßFrancesca RossiSaranshu SinglaFrancesco BimboSatoshi MigitaFrancesco CaddeoSekar VijayakumarFrancesco PirainoSeong Jung KwonFrancisco Eroni Paz Dos SantosSergei TarasovFrancisco Fernández-CamposSergejs GaidukovsFrançoise ChuburuSerwan RafiqFrank AlexisSeung Hun LeeFred BurpoSevil Savaşkan YilmazFrédéric ChaputSeyed Ehsan EndermiFrédéric DelbecqShahin HomaeigoharFuat TopuzShahrokh GhobadlooFu-Gen WuShakeel AhmedFujian TangShantanu PradhanFuyin HsuShaoting LinFu-Yin HsuShaozhi FuGábor KatonaSharafaldin Al-MusawiGabriel Goetten De LimaSharif Md AbuzarGabriela DorciomanShashank GorityalaGabriela IonitaShazid Md. SharkerGabriele DomenicoSheeja Saji VargheseGaelle RoudautShih-Feng ChouGaetano GiammonaShijian WuGaetano IsolaShikha AgarwalGandhi Rádis-BaptistaShikha AwasthiGanesh IngavleShim Sung LeeGarry KerchShinji SakaiGary WnekShu-Chuan LiaoGebremariam BirhanuShuhua HanGeorgios BokiasShujahadeen B. AzizGerald S. ManningShuko SuzukiGerardo BurtonShuquan ChangGerardo PrietoSibel İlbasmiş TamerGhasem SargaziSiddharth GautamGianCarlo PecorariSilvano GeremiaGianpaolo SerinoSilvia BarbonGideon RotichSilvia TampucciGiorgio GrilloSimona CăprărescuGiovanna Rossi-MárquezSimona SennatoGislaine Ferreira NogueiraSimone S. SilvaGiulia Di PrimaSitakanta SatapathyGiulia RonchiSiu Hong Dexter WongGiuseppe LazzaraSizhao ZhangGloria Huerta-AngelesSlawomir KadlubowskiGopakumar GopalakrishnanSnezana Ilic-StojanovicGratiela Gradisteanu PircalabioruSolmaz HajizadehGraziele Grossi Bovi KarataySon NguyenGrzegorz KowalskiSondes GargoubiGrzegorz MakomaskiSoodabeh DavaranGuangxue ChenSreekar Babu MarpuGuijun WangSrini RaghavanGuoliang YingStefan-Ovidiu DimaGustavo César DacanalStephen C MorattiGuy GartyStephen InbrajGuya Diletta MarconiStoyan GutzovGyorgy SzekelySudip K. PattanayekGyo-woo LeeSu-Mi HurHadi RastinSung-Hwan JangHadi SamadianSupachok TanpichaiHaitao YuSwarup RoyHaiyi LiangSyed Haroon KhalidHamada ElwanSyed ShahabuddinHanna KastnerT. IvanovaHassan Karimi-MalehTae-Jong YoonH.balaji RagavendraTahir KhurooHeather A. ColburnTahira PirzadaHelena KellyTakashi MiyataHenri VahabiTakehiko GotohHenry Chun Hin LawTakuji YamamotoHimanshu KathuriaTakuma KurehaHiroaki SaiTangirala Venkata Krishna KarthikHiroshi NonakaTanja JurkinHiroto OzakiTannin SchmidtHiroyasu YamaguchiTao Lin SunHiroyuki IjimaTarek AhmedHitoshi TonomuraTasuku NakajimaHong Chul MoonTatiana EliseevaHongliang LiuTeresa CerchiaraHongshun YangThais L. T. Da SilvaHongxiang HuThashree MarimuthuHorst Henning WinterThomas J BoydHossam E. EmamThomas M ZydowskyHuan WangTianyu ZhuHui LuoTilahun Ayane DebeleHui ZhangTing WangHuifang XuTomasz RebisIciar AstiasaranTomoki MaedaIkram Ullah KhanTomonari SumiI-Ming ChuTomonari TanakaInderbir SinghToshiaki DobashiInimfon UdoetokToshihisa TanakaIntan Rosalina SuhitoToshikazu TakigawaIoana DemetrescuTran Ngoc QuyenIoanna MandalaTrichet LeaIoannis A. AsimakopoulosTzu Hsuan ChiangIoannis K. KarabagiasU Gianfranco SpizzirriIolanda De MarcoUlises Gomez-PinedoIrene Lara-SáezUrsula WindbergerIrina Elena RaschipUsman MinhasIrina SavinaVaclav SlovakIsabelle PochardVahid MorovatiIsma Liza Mohd IsaValery V. KonshinIsopencu GabrielaVanda SerraIstván AntalVasyl M. HaramusIuga MadalinaVegar OttesenIvan S. ChaschinVenkata ChevaliJacek NowaczykVera V. BudaevaJack DouglasVeronika PavliňákováJack HarrowfieldVictor EremeyevJakub M. GacVijay KumarJamal UddinViktor I. SevastianovJan LabutaViktor Korzhikov-VlakhJason T.C. TzenVineet RaiJasvir DalalVittorio ChecchiJatupol JunthipVladimir GegechkoriJaved AliVladimir LozinskyJay RamapuramWansu ParkJean-Jacques BonventWanyu ChenJean-Paul Jay-GerinWei HanJennifer J. GuerardWei ShaoJennifer PattersonWei YuJens GaitzschWei-Chia SuJi Hyun RyuWei-Chun ChinJi LiuWei-Dong HeJia AnWeijun ZhangJiacan SuWeilue HeJiancheng LaiWeiren ChengJianyong ZhangWeiyi SuJin-Feng XingWenbo HanJing LiuWen-Chao LiuJingjing YouWenguo CuiJiri SmilekWenjiang LiJi-Won ParkWenjing SongJoana C. AntunesWen-Yu SuJoana RoloWuge BriscoeJoanna CichowskaWujie ZhangJoanna Skopinska-WisniewskaXiang LiJoanna ZemłaXiaodeng YangJoão Paulo BorgesXiao-Feng SunJohann P. KlareXiaoliang QiJohanna CastañoXiaomin TangJohn BartlettXiaoning ZhangJohn L. DaristotleXiaoqiang XueJolanta LiesieneXiao-Wei ChenJonathan SteedXiaoxia LeJong Hwa JungXin DuJorge Alegre-CebolladaXinda LiJorge Cesar MasiniXing WangJosé A. MartinsXuan ZhangJosé López CastroXuanrong SunJose Luis Gerardo-NavaXun SongJosefina PonsYakov LapitskyJoshua S BoatengYan HanJothi VargheseYan Tao ChenJózef HaponiukYan ZubavichusJózsef KalmárYanchuan GuoJuan Manuel Lázaro-MartínezYang HuJuan Pedro FernándezYan-jie WangJukka KetojaYasumitsu MatsuoJules MagdaYasuyuki MakiJulian L. GendreauYi CaoJulian OberdisseYifeng CaoJuliette FitremannYifeng LeiJuliusz A. WolnyYilin ZhangJun ChenYi-Ping FangJun MeiYishu YanJun WuYi-Ting ChiangJunbo HeYoichiro MoriJung Seung LeeYoko IwamotoJun-ichi KadokawaYonghang XuJun-Xia XiaoYonghou XiaoJustin C. BurrellYongjun MenKadir OzaltinYoshinori TakashimaKajal GhosalYosra ToumiaKamol DeyYoun Soo KimKampanart HuanbuttaYoung-Ho ChoKar Wey YongYoungho EomKarolina PyciaYu. F. ZuevKarthikeyan VenkatachalamYuan JiangKatarzyna KlimekYuan XuKatarzyna Marciniak-LukasiakYuichi OyaKatarzyna Marciniak-ŁukasiakYujen ChouKathryn Taylor-PashowYun Jung HeoKatyayani TatipartiYuping WangKawee SrikulkitYuriy MalyarKe QuYury A. SkorikKęstutis BaltakysYuta YoshizakiKevin CavicchiYutaka OhsedoKevin DzoboZahoor FarooqiKhaled ElsaidZahra BagheriKhaled GiasinZeeshan TariqKhaled SaoudZhaogang YangKhalid RabaehZhaoyang DingKinga KorniejenkoZhexenbek ToktarbayKlaudia KaniewskaZhihong ZhangKohzo ItoZhongbin PanKrishna K DamodaranZulamita Zapata-BenabitheKulwinder KaurZulhelmi Alif Abd HalimKummara Madhusudana Rao



